# The Role and Importance of Using Sensor-Based Devices in Medical Rehabilitation: A Literature Review on the New Therapeutic Approaches

**DOI:** 10.3390/s23218950

**Published:** 2023-11-03

**Authors:** Dan Alexandru Szabo, Nicolae Neagu, Silvia Teodorescu, Mihaela Apostu, Corina Predescu, Carmen Pârvu, Cristina Veres

**Affiliations:** 1 Department of Human Movement Sciences, George Emil Palade University of Medicine, Pharmacy, Science, and Technology of Targu Mures, 540139 Targu Mures, Romania; nicolae.neagu@umfst.ro; 2 Department ME1, Faculty of Medicine in English, George Emil Palade University of Medicine, Pharmacy, Science, and Technology of Targu Mures, 540139 Targu Mures, Romania; 3 Department of Doctoral Studies, National University of Physical Education and Sports, 060057 Bucharest, Romania; silvia.teodorescu@unefs.ro; 4 Department of Special Motor and Rehabilitation Medicine, National University of Physical Education and Sports, 060057 Bucharest, Romania; mihaelaapostu@yahoo.com (M.A.); corina.predescu@yahoo.com (C.P.); 5 Faculty of Physical Education and Sports, “Dunărea de Jos” University, 63-65 Gării Street, 337347 Galati, Romania; carmen.parvu@ugal.ro; 6 Department of Industrial Engineering and Management, University of Medicine, Pharmacy, Science and Technology of Targu Mures, 540142 Targu Mures, Romania; cristina.veres@umfst.ro

**Keywords:** medical rehabilitation, sensor-based devices, virtual reality in rehabilitation, rehabilitation robotics, artificial intelligence

## Abstract

Due to the growth of sensor technology, more affordable integrated circuits, and connectivity technologies, the usage of wearable equipment and sensing devices for monitoring physical activities, whether for wellness, sports monitoring, or medical rehabilitation, has exploded. The current literature review was performed between October 2022 and February 2023 using PubMed, Web of Science, and Scopus in accordance with P.R.I.S.M.A. criteria. The screening phase resulted in the exclusion of 69 articles that did not fit the themes developed in all subchapters of the study, 41 articles that dealt exclusively with rehabilitation and orthopaedics, 28 articles whose abstracts were not visible, and 10 articles that dealt exclusively with other sensor-based devices and not medical ones; the inclusion phase resulted in the inclusion of 111 articles. Patients who utilise sensor-based devices have several advantages due to rehabilitating a missing component, which marks the accomplishment of a fundamental goal within the rehabilitation program. As technology moves faster and faster forward, the field of medical rehabilitation has to adapt to the time we live in by using technology and intelligent devices. This means changing every part of rehabilitation and finding the most valuable and helpful gadgets that can be used to regain lost functions, keep people healthy, or prevent diseases.

## 1. Introduction

It has been observed in recent years that remarkable progress has been made in the development of sensors. These advancements in sensor technology provide opportunities that have never been available before for the early diagnosis and prevention of human diseases by detecting critical biomarkers and health assessments via monitoring and analysing human physiological signals in healthcare and biomedical applications [1].

The development and integration of materials science, sensing methods, wireless technologies, and the Internet of Things (I.o.T.) have considerably helped the evolution of wearable gadgets. Wearable technology has emerged as an innovative new approach to leading a healthy lifestyle. The amount of wearable technology worldwide has been on the rise, as shown by research conducted by the International Data Corporation (I.D.C.) [2]. Over the course of the last two years, it has seen remarkable expansion. Even though the pandemic hit it in 2020, the company’s exports nonetheless climbed by 32%, reaching a total of 444.7 million CNY. It is clear that wearable technology is continuing to be popular, and there is also a rising demand in the market [3]. Wearable technology for health monitoring often consists of miniature rigid circuit boards and block power sources attached to different regions of the human body, most notably the wrist, to monitor physiological data in real-time [2].

Wearable gadgets, also known as devices that can be worn on the body and monitor many activities and characteristics, are becoming more popular among the general public and are seeing increased sales and use. Wearable technology has many applications, but one of the most important ones is in medicine, namely, in biomedical research, clinical treatment, personal health practices and monitoring, technological development, and engineering. In this setting, the use of wearables for medical purposes has been linked to several promises and advantages for more digital, individualised, preventative care [4]. 

The availability of consumer and medical products that use wearable sensor technology has progressively increased over the last several years. This includes a broad range of well-established consumer products. Wearable technologies can offer real-time feedback on a person’s health status. As a result, they may provide an objective alternative to manage and monitor the course of chronic diseases, such as in the case of the elderly, within rehabilitation, and for people who suffer from various impairments. Because of its hardware capability, compact size, and reduced cost compared to analogous medical tools capable of monitoring the same vital signs, wearable sensors have widespread use in healthcare [5]. In addition, wearable technology enables rehabilitation outside of the hospital in an ambulatory setting, which brings the overall cost of intensive therapy down [6].

The research community and the industry are using I.o.T. applications with real-time integrated devices to improve the lives of average users. The I.o.T. is increasingly used everywhere, especially in the healthcare system. This is because the healthcare system offers clinical facilities, nursing for patients, cutting-edge searching and monitoring of medical challenges, computer-based treatment, and constant backup facilities for patients [7].

With the evolution of body area sensing and network technologies, wearable rehabilitation technology has opened up the possibility of independent training, which has many advantages over traditional rehabilitation services. Inertial measurement units (IMUs), including accelerometers and gyroscopes, have been widely used in technology-assisted rehabilitation with sufficient effectiveness. However, despite the potential use of IMU-based sensors in neurorehabilitation and for treating musculoskeletal impairments, some such sensors have been used in clinical trials [8].

Image-based and wearable sensor systems have been used to assess exercise and body movements, applying methods developed in human activity recognition. Image-based systems have many challenges (related to configuration, line of sight, and computational requirements) that may limit their suitability for home rehabilitation assessment and posture monitoring. Portable sensors with inertial measurement units (IMUs) have been widely used in various scenarios. IMUs are easy to incorporate, compatible with a variety of contexts, and present fewer privacy problems. This device seems to be a potential solution for monitoring adherence to rehabilitation protocols and posture [9,10].

This study aims to analyse the role and importance of using sensor-based devices in medical rehabilitation, evaluating their effects on the quality of patients’ rehabilitation, as well as the pact of using smart devices on patients and physiotherapists. Also, this study aims to evaluate the use of advanced technology in medical rehabilitation and its usefulness at the expense of classical methods and techniques used since ancient times by physiotherapists.

The degree of novelty of this review consists of addressing a current issue in physical therapy, namely, the use of smart devices based on sensors on the quality of rehabilitation of various pathologies and dysfunctions of patients. Their use nowadays has become a beneficial and frequently used alternative, both due to the pandemic context we have gone through and the accelerated advancement of technology.

## 2. Materials and Methods

Using the databases PubMed, Web of Science, and Scopus, the current literature review was compiled between October 2022 and February 2023 in compliance with the P.R.I.S.M.A. guidelines (Table 1).

EndNote X9, a reference management application, was used to construct the records recognised from the databases using the abovementioned keywords. It was also used to help delete articles that were duplicated.

After that, depending on the designs of the studies, we attempted to include all articles of the following types: a systematic review, a meta-analysis, a case–control study, a cross-sectional study, a literature review, and a case report. However, we did not include expert opinions, letters to the editor, or conference reports.

The data were obtained via the use of a word form. In order to develop the current literature review, we went through each article that was chosen for evaluation and extracted the material that we felt was relevant to the respective sub-chapter.

We want to mention the high number of duplicate articles because many are found in two or even three databases. 

**Figure 1:**
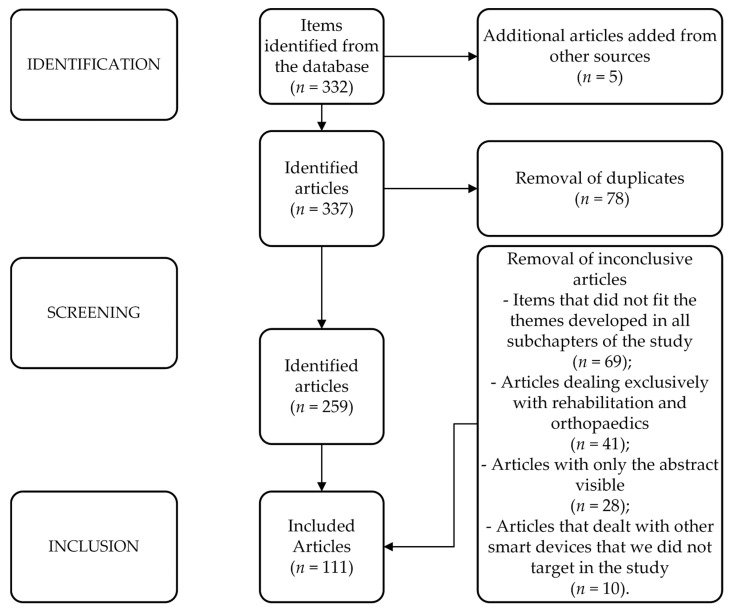
Flowchart for the selection of studies based on the P.R.I.S.M.A. criteria.

The first search turned up 337 different titles in the databases mentioned above; the database software deleted 78 duplicate articles, and 2 more articles were brought in from external sources. After performing a relevancy check on the titles and abstracts of the remaining 259 papers, we eliminated 78 research papers. At the screening stage, studies were eliminated because of items that did not fit the themes developed in all subchapters of the study (*n* = 69), because the articles dealt exclusively with rehabilitation and orthopaedics (*n* = 41), because only the abstracts of 28 articles were visible, and because 10 articles dealt exclusively with other sensor-based devices and not medical ones; the inclusion phase resulted in 111 articles being included in the study. The complete P.R.I.S.M.A. diagram is seen in Figure 1.

## 3. Medical Rehabilitation: New Therapeutic Approaches

According to the World Health Organization, physical rehabilitation is a set of interventions to optimise the body’s functions and reduce the disability of people with various health problems [11]. Due to the awareness of the importance of functional and medical rehabilitation, rehabilitation services are continuously increasing, as well as chronic conditions and disabilities [10]. Medical rehabilitation aims to improve the quality of life, ensuring, in addition to the acquisition of functional independence, the individual’s reintegration into society, one of the main objectives established at the beginning of a rehabilitation program [12].

Physical therapy, also known as physiotherapy (which comes from the Greek words fysis, which means nature, and therapia, which means treatment), combines a number of different factors in order to treat and prevent diseases. These factors include natural elements (the sun, the sea, healing mud, water, and movement) and manufactured elements (electric current, ultrasound, artificial light, laser rays, and magnetic field). The effects of their action include a reduction in pain, stimulation of restoration processes, increased range of motion, activation of immunological systems, and improved biochemical performance. When compared to other treatments, physical therapy is not only less expensive but also less intrusive and more straightforward to put into practice [13,14,15].

Physicians such as Hippocrates, and later Galen, are believed to have been the first physiotherapy practitioners. Around 460 BC, some practitioners advocated using massage, manual therapy methods, and hydrotherapy to treat individuals. Due to the creation of orthopaedics in the 18th century, devices such as the Gymnasticon were first designed to treat gout and other ailments of a similar kind through systematic joint exercise, similar to later developments in physiotherapy [16].

**Figure 2:**
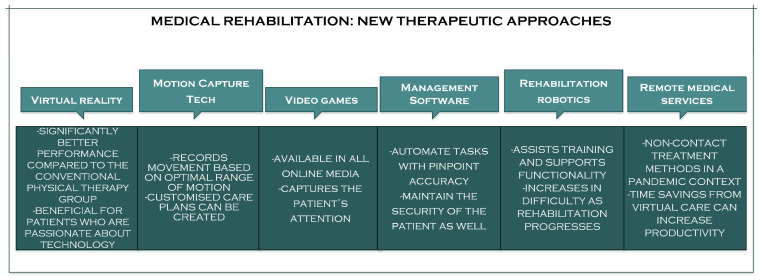
The new therapeutic approaches in medical rehabilitation.

Various factors impair a person’s physical abilities; many stem from a muscle, an accident, a surgical procedure, degenerative diseases, cardiovascular disease, and ageing [17]. Neurological and orthopaedic dysfunctions (acquired deficiency, congenital deficiency, or incorrect posture) can lead to mobility issues [18]. Rehabilitation is necessary to maintain or restore the patient’s mobility or function. The goal is to recover these impaired functions entirely and, if that is not feasible, to enhance the movement of the upper and lower extremities responsible for locomotion in order to attain functional independence [19,20]. In recent years, physical therapy office owners have seen technological trends that could permanently change the standard of patient care. Because of these recent advancements in physical therapy, patient treatment is now more readily available, simpler to deliver, and provides better outcomes than a few industry standards that have been “tested and proven” (Figure 2) [21].

### 3.1. Virtual Reality

As a result of recent technology breakthroughs, virtual reality (V.R.) is becoming an increasingly popular tool for use in healthcare settings. These improvements present opportunities for diagnosis and therapy. V.R. is a technology that creates the appearance that the user is physically present in a simulated environment by means of the usage of a headset to mimic a reality in which the user is immersed in a simulated setting. V.R. provides users with a wide variety of options for how they may engage with a virtual environment or with virtual characters. A better feeling of realism and the ability to have meaningful interactions may be provided to the user through virtual characters, also known as avatars [22].

The term “virtual reality” refers to a computer-generated, three-dimensional virtual environment that users are able to interact with. A user’s many senses will be engaged by the technology, which will then enable the user to interact with realistic 3D virtual surroundings. V.R. is a kind of extended reality technology that differs from augmented reality (A.R.), which is another sort of extended reality technology. Whereas AR superimposes digital data onto the actual world, V.R. blocks off the real world and allows interaction with a simulated virtual environment. Delivering simulated virtual worlds may either be achieved in a non-immersive way or in an immersive way. In non-immersive virtual reality, the virtual world is implemented by projecting it onto a big display or wall screen (for example, Powerwall displays and cave automated virtual environments). On the other hand, in immersive virtual reality, a head-mounted display is often used to offer complete immersion and interaction with the virtual environment [23].

### 3.2. Motion Capture Tech

Motion capture (Motion Capture Tech) refers to the process of documenting movement, whether of people or things. It has applications in the armed forces, the entertainment industry, sports and medicine, computer vision, and robot validation applications [22].

Tracking the mobility of human bodies is now one of the study fields that are seeing the highest growth. The word “motion capture” (MoCap) has been defined in various ways by various researchers, each according to the particular study domain they specialise in. The process of identifying and establishing the nature of a patient’s illness or condition is known as medical diagnosis. This process, which plays an essential part in the area of healthcare, is essential. Historically, diagnosing a medical condition mainly depended on the knowledge and experience of experts working in healthcare. Nevertheless, there has been a dramatic shift in how diagnoses are made in recent years due to technological developments and the rise of artificial intelligence (A.I.) and Motion Capture Tech (MCT). A medical diagnosis based on A.I. and MCT uses machine learning algorithms to examine massive volumes of patient data, such as medical records, imaging scans, and genetic information, to aid medical practitioners in making accurate and fast diagnoses [24].

### 3.3. Video Games

In the last five years, for instance, mobile gaming has experienced exponential growth in every country, accompanied by the appearance of powerful processors with stunning graphics and high-speed performance. These technological advancements guarantee that each patient possesses the necessary equipment for the physical therapist to prescribe highly engaging treatment plans [25].

In light of this, recent advancements in gaming technology and telerehabilitation are shifting the proportion of a therapist’s time spent on motor practice as opposed to behavioural intervention. This innovative form of therapy allocates most of the therapist’s time to behavioural interventions to improve arm usage. These interventions may be carried out remotely through telerehabilitation, which increases accessibility. Patients may use gaming technology designed for rehabilitation to manage their own intense motor practice at home. The games increase in complexity over time, provide quick feedback, and keep track of patients’ progress. This reversed approach to patient care was shown to be risk-free and doable, and the patients favoured it. However, in order to evaluate whether or not dedicating therapist time almost exclusively to behavioural intervention may be just as beneficial as typical rehabilitation, which predominantly employs therapist time for motor practice, a definite pragmatic randomised controlled study is still required. In addition, this self-managed, time-efficient strategy’s success must be compared with the efficacy of a complete time-intensive intervention (CI therapy), in which a therapist gives both motor practice and behavioural treatment to the patient [25].

### 3.4. Physical Therapist Practice Management Software

In this demanding era of usage, outcomes research, and cost efficiency, it is necessary to have open-ended medical practice management systems that are efficient with both time and resources and have the ability to enhance patient care. As a result, medical practice offices in developing nations are going through significant micro-processing shifts, a crucial standard for recording and conveying patient care that has emerged over the last decade.

The patient management system (P.M.S.) and the electronic medical record (E.M.R.) are currently considered the most important types of medical software in emerging nations’ medical informatics. An electronic medical record (E.M.R.) is a repository of information regarding the health of a subject of care that is in a form that a computer can process, can be stored and transmitted in a secure manner. On the other hand, medical practice management software, often known as P.M.S.s, is a form of medical software used to manage the day-to-day activities in a hospital, physical therapy at home, or doctors’ clinic. This is accomplished by dealing with the day-to-day operations of a medical practice at a physician’s office [26].

### 3.5. Rehabilitation Robotics

At the moment, robots for rehabilitation are driving a significant amount of research and development, and a great number of exciting new avenues are developing, both directly related to mechanical instruments and in the assistance of a rehabilitation process that is much more extensive.

There is potentially a good number of benefits that may come from providing rehabilitation with the application of mechanical technology. To be more specific, it makes it possible for more rigorous and patient-specific rehabilitation operations and services (which, in turn, increases the amount and trait of therapy that can be administered). In addition, it enables all team members (including physiotherapists, physicians, bioengineers, and others) to establish and administer specific work parameters in order to personalise and optimise the patient’s rehabilitation process (the type of workout, the proportion of contribution from the machine, the power, and the duration of the exercise) [27].

### 3.6. Remote Medical Services

Physical therapists are discussing telehealth in physical therapy more frequently, regardless of whether or not they have a well-known work system or are seeking to operationalise a new telehealth base [28]. As an outcome of the unanticipated repercussions of the coronavirus disease (COVID-19) pandemic, considerable adjustments have been made to the way in which health systems provide healthcare services. Stay-at-home orders, lockdowns, and social distancing are examples of the types of epidemic control measures that have caused disruptions in the continuity of healthcare provision for COVID-19 patients and those whose conditions are not related to COVID-19. One of the adjustment mechanisms includes the increased use of telemedicine to maintain this continuity, as it has appeared to be interrupted by these types of epidemic control measures. As a result of the circumstances in which the advantages of telemedicine have been acknowledged in earlier instances of public health catastrophes, such as the severe acute respiratory syndrome (SARS) or the Middle East respiratory syndrome (MERS) (2), this accomplishment has appeared to utilised, with certain adjustments made for the COVID-19 pandemic [29]. 

Patients who live in distant places, such as rural populations in poor nations, who often have limited access to healthcare, may benefit from the increased availability of healthcare that is made possible via telemedicine. In addition to this, it is able to swiftly deploy large numbers of physicians, which helps with triage and supplies clinical services in situations when health institutions are unable to fulfil demand [30]. Moreover, from a physical therapist’s standpoint, the time decreases associated with virtual care can increase productivity. In general, telehealth technology is gaining traction because the product is superior, quicker, and more time-efficient than in-person therapy in particular use incidents and where this form of medical rehabilitation is feasible [31].

## 4. The Importance and Benefits of Using Sensor-Based Devices in Medical Rehabilitation

**Figure 3:**
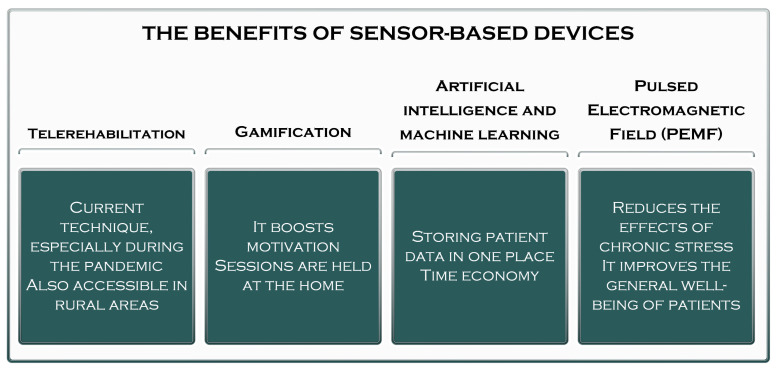
Sensor-based devices—benefits.

The importance and especially the benefits of using sensor-based devices in medical rehabilitation is an issue entirely addressed by physiotherapists and other specialists in the field of rehabilitation; as technology advances at a significantly accelerated pace and devices are created for the rehabilitation of lost functions, the devices have become very complex and require advanced training of the specialists who will use them (Figure 3) [31].

Sensor-based devices appeared relatively recently in medical rehabilitation, incorporating many techniques that physical therapists can use to achieve a series of goals established at the beginning of the rehabilitation protocol [32].

Sensor-based devices appear in many forms and can be used in many ways, aiming to rehabilitate some lost functions [33]. Thanks to specially created software, these devices stimulate the receivers to regain lost or limited functions. To make rehabilitation interactive and as efficient as possible, many applications and software models have been created to increase the quality of rehabilitation [33].

### 4.1. Telerehabilitation

Telerehabilitation represents a topical technique that will be around for a while [34]. With the prevailing pandemic restricting people to their homes, teleconsultation has become an enormous trend, making life extremely convenient for patients and doctors [35].

Patients in rural areas can easily connect with specialists, parents can enjoy physical therapy sessions without dropping their kids off at daycare, and others who struggle to go out/have social anxiety can be treated from the comfort of home [36].

In fact, in countries such as Poland and Germany, telerehabilitation is reimbursed as part of health plans for citizens [37].

### 4.2. Gamification

Gamified rehabilitation applications help to engage patients, which plays a crucial role in deciding the success or failure of treatment [38]. Even the best plans fall apart when the patient fails to perform the movements due to a lack of motivation or, in some cases, boredom [39]. These apps provide an addictive and rewarding experience, releasing dopamine each time the patient enters the game and prompting patients to return and maintain their high mood [40]. Some of these apps allow patients to perform their exercise program at home while playing fun video games. The games involve prescribed physical therapy movements tracked with wearable sensors. Repetitions, trajectories, and time taken are followed accurately, and exercises are performed incorrectly so that the plan can be modified later [40].

### 4.3. Artificial Intelligence and Machine Learning

These two phrases are more than buzzwords, and they can change the image of the healthcare industry [41]. These mean less time spent writing notes and Googling treatment guidelines, recognising similar patterns in patient lesions, and simplifying patient-specific procedures—automatically [42]. At the same time, they aim to store all patient data in one place and be analysed to discover various perspectives we can use to improve our treatment as physiotherapists [43,44,45]. 

### 4.4. Pulsed Electromagnetic Field (P.E.M.F.)

Pulsed Electromagnetic Field P.E.M.F. therapy is a promising solution to reduce the adverse effects of chronic stress –which is highly prevalent today [45]. Physiotherapists can use P.E.M.F. to stimulate cells to improve the overall well-being of their patients [46]. As a large portion of the workforce works from home, their increased exposure to harmful rays and frequencies (cellphones, blue lights, and Wi-Fi) has added to the increased time spent on electronic gadgets, the stress of additional tasks, and the absence of rest, creating a clear need for this type of sensor [47,48].

## 5. The Benefits of Using Sensor-Based Devices on Patients

### The Quality of Rehabilitation of Lost Functions Using Sensor-Based Devices

**Figure 4:**
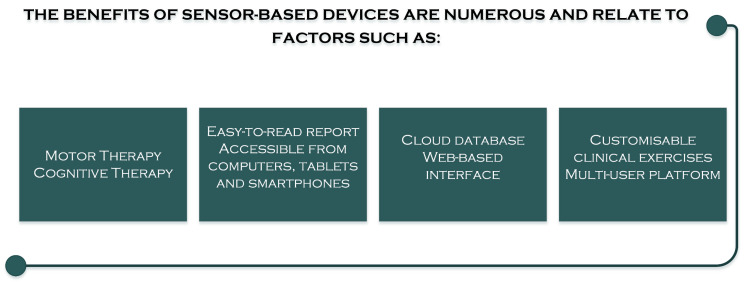
Factors involved in the benefits of sensor-based devices.

Using sensor-based devices has many benefits for patients because the rehabilitation of a missing part represents the achievement of a fundamental objective within the rehabilitation program (Figure 4) [49,50].

Medical devices that use immersive virtual reality use neuro-motor and cognitive rehabilitation techniques for patients of any age (children, adults, and elderly) with neurological disease disorders such as stroke, cerebral palsy, Parkinson’s, and autism [51,52]. Specific medical tools can construct what is known as a “sensory chamber”, which allows the patient to participate in a stimulating, immersive experience that mimics a variety of actual settings. It will enable us to make the patient’s rehabilitation process much more efficient by involving the subject in an exciting experience. It is possible to make changes to exercises in real-time and tailor them to the individual patient’s capabilities [53,54].

Sensor-based systems may generate environments on the walls or floors where the patient can engage with the delivered stimuli. The analysis equipment monitors the patient’s motion and behaviour to adjust the projected environment appropriately. These adjustments include the delivery of powerful incentives and rehabilitative audio–visual feedback via full-body immersion [55]. The program is already set up with a series of workouts, each of which may be adjusted to the amount of difficulty that the user likes, speed of execution and sensitive areas for different categories of patients, and, of course, various pathologies and rehabilitation programs [56]. By using this system, one therapist will be in charge of the rehabilitation of several patients at the same time, who will all be working in parallel [57,58].

Utilising sEMG sensors in addition to IMU sensors to monitor individuals while they exercise is one method used for remote evaluation of the characteristics of rehabilitative exercise described by some authors [59]. The authors noted that the method was conducted on 17 patients undergoing physical therapy, attaining a median reliability of 96% in observing speedup, rotation, angular velocity, and posture statistics during monitored exercises [60]. The developers also mentioned that the method attained this level of precision. A separate study combined sEMG detectors and accelerometers with a game-predicated teaching device and a user-feedback network to create a wearable application. This study’s findings demonstrated the system’s performance, gaming experience, and training impact [61].

Numerous studies [62] have examined the utilisation of force sensors to generate pressure foot assessments. In a different publication, the authors described a portable and discrete system that could calculate ambulatory motion and balancing measurements, such as the deduced centre of mass and the dynamic extremity of equilibrium. The technology can treat impaired motor skills, gait, and equilibrium in Parkinson’s disease, multiple sclerosis, and elderly populations [63]. The absolute root mean square error (R.M.S.) for an ambulatory locomotion and stability system comprising 3D F&M (forces and moments) and IMU sensors was 2.2 0.3 cm. The researchers examined various configurations of pressure sensors on each limb in addition to an ultrasound spectrum estimate and discovered that the R.M.S. error was relatively minor. Their study found that positioning pressure sensors beneath the heel and toe provided a simple and unobtrusive alternative to F&M detection for estimating ambulatory locomotion and dynamic stability [64].

Both Yu et al. [65] and Hayward et al. [66] centred their research on the use of wearable sensor networks for patients who had strokes. The first study investigated whether or not accelerometers may be used as a tool to evaluate real-world upper-limb usage after a stroke, and the second paper analysed the possibility of wider use in clinical and research settings. In the first research study, the authors suggested a remote quantitative Fugl–Meyer evaluation framework for stroke patients. This framework makes use of wearable sensors in order to monitor the movement function of the patient’s upper limbs, wrists, and fingers. In the second study, we investigated whether or not there is a possibility of broad adoption in clinical and scientific settings. These papers highlight the potential of wearable sensor networks to enhance stroke rehabilitation and evaluation, as well as the need for standardised protocols, apps, and data interpretation in order to encourage more deployment of the technology [67].

Integration of small sensor components into on-chip electronic systems with ultra-low power consumption has been made possible by recent technology breakthroughs for the automated detection of functional activities of daily living in patients who have had a stroke. This has led to the creation of “hybrid” wearable sensors, which integrate in a single capsule (i) motion sensing and (ii) E.M.G. detection of muscle activity. These sensors have been made possible because of the rise of hybrid computing. When evaluating and treating patients with motor impairments, hybrid sensors may be especially beneficial for detecting the quality of their movement. In point of fact, evaluating the features of the wearer’s movement and the underlying muscle activity responsible for controlling the movement allows for a more comprehensive analysis of movement dysfunction. An electromyography (E.M.G.) recording component and a motion component, such as an accelerometer or IMU, are included in modern hybrid sensors used for movement monitoring [68,69,70,71].

Roossien et al. (2021) [72] devised a technique for the assessment of lumbar load that is based on the use of sensors. The technique utilises six inertial measurement unit (IMU) sensors placed on the sternum, pelvis, and upper and lower arms. An approach that makes use of artificial neural networks to arrive at an estimate of the net moment that occurs around the L5/S1 intervertebral body is what is used to quantify the lumbar load. In healthy people, the variations in the estimated lumbar load were consistent with the reported intensity levels and the nature of the labour activities, which provided support for the validity of the sensor-based technique. It is possible to utilise this approach to monitor lumbar load in persons with musculoskeletal problems such as lower back pain, evaluate muscle overload during rehabilitation, and assist physicians in customising therapies [73].

Prasanth et al. (2021) [74] conducted a comprehensive study to investigate the sensor-based methodologies that were used for real-time gait analysis. Most of the time, threshold or peak identification techniques are utilised in conjunction with inertial measurement devices attached to the shin and the foot to perform gait analysis. Pathological gait data were used to verify less than one-third of the sensor-based algorithms for gait analysis. Inertial measurement units and rule-based procedures are the ideal options for clinical gait evaluations [73]. This is because both types of methods provide accurate results.

Validated as a Class I medical device, the D.M.D. (Orthelligent knee, OPED, Valley, Germany) [75] is a knee-replacement system. It includes a training program, paper, and online training videos for each exercise, as well as settings for training control for each activity. Additionally, it includes an inertial motion sensor and software that can be downloaded onto individual cell phones. In rehabilitation for problems affecting the hip, knee, and foot (such as injuries or surgical treatments), Orthelligent offers exercises tailored to each step of the process. The sensor of the D.M.D. is a piece of objective measuring equipment that is fastened to the lower leg right below the head of the tibia. After an injury such as replacement of the anterior cruciate ligament (A.C.L.), the D.M.D. is utilised in home-based settings as an add-on to stage-specific routine physiotherapy to conduct particular tests in the categories range of motion (R.O.M.), coordination (motor control), and dynamic testing (strength/speed). Patients have complete control over whatever activities and examinations they choose to participate in. Using the D.M.D. algorithm, measured values of the damaged leg are compared to those of the contralateral unaffected limb. The results of this comparison are then shown as graphs displaying the relative values (symmetry or FIT Index) and changes that occur throughout rehabilitation. The purpose of the Orthelligent system is to encourage patients to carry out the exercises prescribed by their health care practitioner in a manner that is adequate, frequent, and accurate qualitatively, as well as to fortify patients’ motivation via the use of autofeedback in the context of self-monitoring [76].

Monitoring the human body and its dynamics supports exercise, clinical interventions, and physical therapy in terms of patient rehabilitation [77,78]. Based on the information presented in this section regarding the use of sensor-based devices in patient rehabilitation, the use of sensor-based devices in rehabilitation is evident. In order to achieve this objective, inertial sensors, pressure sensors, and bioelectric sensors, particularly those used for electromyography, are frequently combined with bioelectric sensors [76]. Moreover, numerous motion monitoring sensors (such as infrared, ultrasonic, depth sensors, multi-array cameras, and microphones) are typically included [79]. In addition, stimulation systems, such as virtual reality and therapy-adapted video games, are frequently used as supplementary platforms for rehabilitation [80,81].

## 6. The Impact of the Use of Sensor-Based Devices on Physiotherapists

Physiotherapists are specialists in the field of human biomechanics, and they play an essential part in the prevention, diagnosis, evaluation, treatment, and (re)habilitation of persons whose mobility and function are threatened or hindered as a result of ageing, injury, illness, conditions, or environmental factors [82,83,84]. Physical therapists help individuals in all phases of life recover from injuries, decrease pain and stiffness, promote mobility and movement, and maximise function and quality of life by considering a person’s physical, psychological, emotional, and social well-being [85,86]. Physiotherapists work in the health care system, including hospitals, schools, private clinics, home care, long-term care facilities, and organisations [86,87]. The increased need for human resources is a current issue in this field, primarily due to the shift from treatment-focused care to preventive care and the increase in the number of older people with chronic conditions and the associated demands on the healthcare system [88].

In a multidisciplinary team, physiotherapists can work individually with patients with musculoskeletal and neurological conditions, provide fall prevention, and educate patients and caregivers about preventing and managing chronic diseases [89]. Physiotherapists also play a significant part in group rehabilitation programs, which are geared at the prevention and management of chronic illnesses, as well as the promotion of health and well-being in the community [90]. Physiotherapists are also responsible for regaining patients’ physical and functional independence, one of the first goals set at the beginning of a rehabilitation plan [89,91]. At the same time, physiotherapists play a significant role in the area of prophylaxis, as they are the ones who establish prevention programmes for certain diseases and determine their progression and adaptation [92,93]. 

The impact of using sensor-based devices on physiotherapists and specialists in rehabilitation medicine has been discussed and analysed from several perspectives over time [94]. As well as other intelligent devices that have recently appeared and are used in the medical rehabilitation of patients, sensor-based devices considerably facilitate the work of a physiotherapist [95].

As a primary benefit of using sensor-based devices on physiotherapists, we mention the reduced therapy time; many recovery goals are achieved with the help of these devices, shortening both the recovery period of lost functions and the duration of a recovery session [96,97]. The physiotherapist can also work with multiple patients simultaneously using various sensor-based devices, with the physiotherapist monitoring the activity in the physiotherapy office and the effectiveness of the chosen treatment [98]. 

Regarding telerehabilitation and robot-assisted rehabilitation, physiotherapists may face several issues related to the need for a team, as these devices can be coordinated and guided by a reliable specialist [99]. This can be considered both an advantage and a disadvantage for medical rehabilitation specialists [100].

## 7. Limitations of Using Sensor-Based Devices in Medical Rehabilitation

Using sensor-based devices in physical therapy has become increasingly popular [101]. Although the use of sensor-based devices facilitates in many ways both the functional rehabilitation of the patient and the physical work of the physiotherapist, their use has several limitations [102]. The ever-increasing use of devices in physical therapy offices makes physical therapy much more interactive, thus increasing the quality of patients’ rehabilitation [103].

However, the availability of these sensor-based devices is quite limited because they are expensive, most of them being quite challenging to acquire or requiring advanced training of physiotherapists in their correct and effective use [104,105].

Although the benefits of using sensor-based devices in medical rehabilitation have been demonstrated, their use in rehabilitation and rehabilitation programs is still in its infancy, with many physiotherapy specialists opting for classical methods and techniques over advanced technology [105,106].

At the same time, the use of sensor-based devices also has several limitations for physical therapists [107]. Although these devices facilitate the work of physiotherapists, the advancement of technology in rehabilitation medicine forces specialists to keep up with the rapid growth of new methods and techniques, adapting to new demands [108]. Also, these devices, as well as many other robotic devices in the field of physiotherapy, can replace the work of a physiotherapist, so the demand for specialists becomes slightly limited; some attributions and roles of physiotherapists can be covered by a robot or artificial device [109].

Regarding the rehabilitation of the elderly, the use of sensor-based devices may be limited by their willingness to try this type of therapy [110]. At the same time, geriatric patients can find it much more difficult to adapt to rehabilitation and rehabilitation techniques that use these types of devices, being reluctant to follow this type of physical therapy and opting for a classic rehabilitation program, which is sometimes not as practical [111].

## 8. Conclusions

The use of sensor-based devices today represents a highly beneficial alternative to the rehabilitation of lost functions, facilitating both the work of physiotherapists and maintaining the active attention of the patient during a rehabilitation session. Sensor-based devices help patients’ functional rehabilitation thanks to software specially created by specialists, but also by creating interactive activities, adopting a new approach to medical rehabilitation, and using advanced technology, which keeps patients’ attention throughout a rehabilitation session.

The increasingly accelerated advancement of technology makes the field of medical rehabilitation adapt to the era in which we live, based on technology and smart devices, adapting every aspect of rehabilitation and discovering the most valuable and beneficial devices that can be used for regaining some lost functions, maintaining well-being, or preventing some pathologies.

The use of sensor-based devices in medical rehabilitation is undoubtedly one of the current therapies used more and more often in rehabilitation programs and physiotherapy clinics, having numerous benefits both in the restoration of lost functions and in maintaining the active attention of patients, facilitating the work of physiotherapists.

## Figures and Tables

**Table 1:** The search strategy used in PubMed, Web of Science, and Scopus, with each search term.

Database	Search Terms
PubMed	(Sensor-based devices) AND (Rehabilitation) AND (Medical) OR (Smart devices) AND (Rehabilitation) AND (Medical) OR (Video Games) AND (Rehabilitation) AND (Medical) OR (Rehabilitation robotics) OR (Artificial intelligence) AND (Rehabilitation) AND (Medical) OR (Virtual reality) OR (V.R.) AND (Rehabilitation) AND (Medical) OR (Medical rehabilitation) AND (New therapeutic approaches) OR (Physical therapy) AND (Software) OR (Medical rehabilitation) AND (Software) OR (Gamified rehabilitation).
Web of Science	(Virtual reality) OR (V.R.) AND (Rehabilitation) AND (Medical) OR (Medical rehabilitation) AND (New therapeutic approaches) OR (Physical therapy) AND (Software) OR (Medical rehabilitation) AND (Software) OR (Gamified rehabilitation) OR (Sensor-based devices) AND (Rehabilitation) AND (Medical) OR (Smart devices) AND (Rehabilitation) AND (Medical) OR (Video Games) AND (Rehabilitation) AND (Medical) OR (Rehabilitation robotics) OR (Artificial intelligence) AND (Rehabilitation) AND (Medical).
Scopus	(Video Games) AND (Rehabilitation) AND (Medical) OR (Rehabilitation robotics) OR (Artificial intelligence) AND (Rehabilitation) AND (Medical) OR (Virtual reality) OR (Sensor-based devices) AND (Rehabilitation) AND (Medical) OR (Smart devices) AND (Rehabilitation) AND (Medical) OR (V.R.) AND (Rehabilitation) AND (Medical) OR (Medical rehabilitation) AND (New therapeutic approaches) OR (Physical therapy) AND (Software) OR (Medical rehabilitation) AND (Software) OR (Gamified rehabilitation).

## Data Availability

Not applicable.
